# A Brief Review of Bioactive Metabolites Derived from Deep-Sea Fungi

**DOI:** 10.3390/md13084594

**Published:** 2015-07-23

**Authors:** Yan-Ting Wang, Ya-Rong Xue, Chang-Hong Liu

**Affiliations:** State Key Laboratory of Pharmaceutical Biotechnology, School of Life Science, Nanjing University; 163 Xianlin Avenue, Nanjing 210023, Jiangsu, China; E-Mails: wangyanting2012@qq.com (Y.-T.W.); xueyr@nju.edu.cn (Y.-R.X.)

**Keywords:** deep-sea fungi, bioactive compounds, anticancer, antimicrobial, antiviral, antifungal

## Abstract

Deep-sea fungi, the fungi that inhabit the sea and the sediment at depths of over 1000 m below the surface, have become an important source of industrial, agricultural, and nutraceutical compounds based on their diversities in both structure and function. Since the first study of deep-sea fungi in the Atlantic Ocean at a depth of 4450 m was conducted approximately 50 years ago, hundreds of isolates of deep-sea fungi have been reported based on culture-dependent methods. To date more than 180 bioactive secondary metabolites derived from deep-sea fungi have been documented in the literature. These include compounds with anticancer, antimicrobial, antifungal, antiprotozoal, and antiviral activities. In this review, we summarize the structures and bioactivities of these metabolites to provide help for novel drug development.

## 1. Introduction

Fungi are well known for their vast diversity of secondary metabolites, which include many life-saving drugs and highly toxic mycotoxins [1]. Deep-sea fungi are the fungi that inhabit the sea and its sediment at a depth of over 1000 m below the surface [2]. Although the conditions in deep-sea environments are extreme and can be characterized by the absence of sunlight irradiation, predominantly low temperature, high hydrostatic pressure, and oligotrophy, it has been reported that fungi are abundant and diverse in these environments [3,4,5]. According to literature surveys, the first documented deep-sea fungi were isolated from the Atlantic Ocean at a depth of 4450 m approximately 50 years ago [6]; however, it was not until 2006 that the first bioactive metabolite of the deep-sea fungus *Chromocleista* sp. was described by Park *et al.* [7]. After that, many biologically active secondary metabolites have been isolated from deep-sea fungi and evaluated for their activities against cancer, pathogenic fungi, bacteria, virus, and larval settlement [1,7,8,9]. Many bioactive metabolites have shown potential as an excellent resource for the discovery of new drugs [10,11]. The intent of this review is to summarize the new and/or bioactive compounds produced by deep-sea fungi.

## 2. Diversity of Deep-Sea Fungi

Although the environmental conditions in the deep sea cannot support many organisms, it is now well recognized that the deep sea is home to rich and diverse microbial communities [12]. Apart from bacteria and archaea [13,14,15], fungi in deep-sea environments have been extensively studied [5,16,17] in the past 50 years since the isolation of the first reported deep-sea fungi from the Atlantic Ocean at a depth of 4450 m [6]. The investigated deep-sea environments include the Gulf of Mexico [12], the Mariana Trench (11,500 m) [18], the Chagos Trench (5500 m) [19], the Central Indian Basin (5000 m) [17], the South China Sea [20,21,22,23], the Antarctic Ocean [24,25], the Eastern Mediterranean [26], the Pacific Ocean [27], the Black Sea [28], the North Pacific Ocean [29], the East Indian Ocean [30], and the Central Arabian Sea [31]. For instance, Xu *et al.* [32] have described 175 deep-sea fungi that were isolated from 15 sediments in the Eastern Pacific Ocean, the South Atlantic Ocean, and the Southwest Indian Ocean. These fungi, including 93 yeast and 82 filamentous fungi, belonged to 17 genera: *Rhodosporidium*, *Rhodotorula*, *Aspergillus*, *Cladosporium*, *Penicillium*, *Alternaria*, *Fusarium*, *Acremonium*, *Phoma*, *Tritirachium*, *Chaetomium*, *Exophiala*, *Engyodontium*, *Sistotrema*, *Schizophyllum*, *Tilletiopsis*, and *Hormonema*. Most of the fungi were either isolated using culture-dependent method or proved based on sequence analysis of the ribosomal RNA gene, and are classified into Ascomycota and Basidiomycota. Particularly, *Aspergillus* and *Penicillium* belonging to Ascomycota are dominant in deep-sea environments [32,33].

Along with the development of modern instruments and techniques used for sampling and researching, more and more deep-sea fungi have been collected [20,22,34,35,36,37,38]. The rich and diverse communities of deep-sea fungi increase the pool of fungi available for natural bioactive product screening and new drug discoveries.

## 3. Bioactive Metabolites of Deep-Sea Fungi

### 3.1. Anticancer

With the changes in the living environment, cancer has become one of the major causes of death worldwide [39]. Many techniques have been applied to control cancer, such as surgery, radiotherapy, and chemotherapy. Out of all these techniques, chemotherapy is the most commonly used and the most effective method to treat cancer so far. However, the application of chemotherapeutic agents has been greatly restricted because most of them have high cell toxicity and provoke severe adverse reactions in human beings [40]. Therefore, seeking for high-efficiency, low-toxicity anticancer agents from deep-sea fungi has become one of the research subjects in the current pharmacy field.

#### 3.1.1. Polyketides Compounds

**Figure 1:**
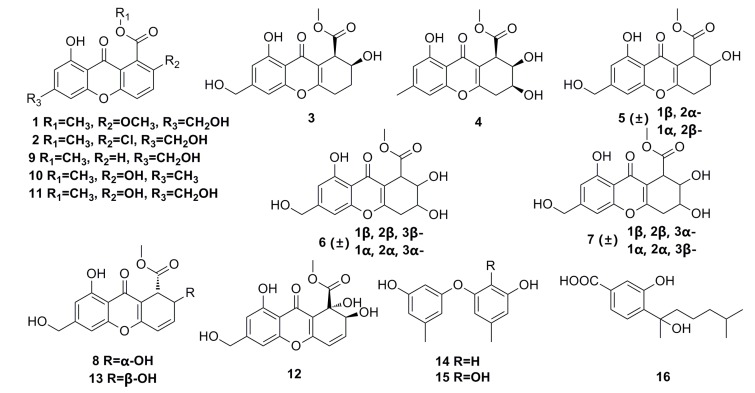
Chemical structures of compounds **1**–**16**.

Eight new chromones, engyodontiumones A–H (**1**–**8**), and eight known polyketides (**9**–**16**) (Figure 1) have been isolated from the deep-sea fungus *Engyodontium album* DFFSCS021. These polyketide compounds show a significant selective cytotoxicity against human histiocytic lymphoma U937 cell line with IC_50_ of 4.9–8.8 μM. In addition, compounds **8**, **12**, and **13** exhibit mild antibacterial activity against *Escherichia coli* and *Bacillus subtilis*, and compound **12** shows potent antilarval activity against barnacle *Balanus amphitrite* larval settlement [41].

**Figure 2:**
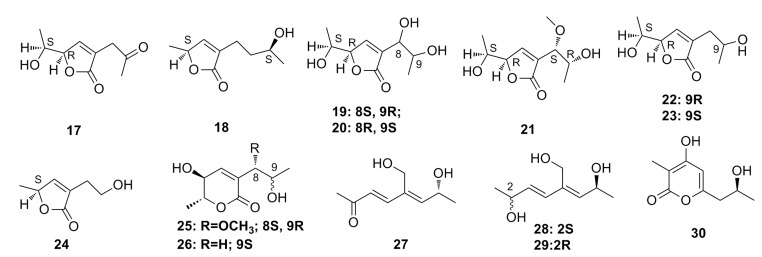
Chemical structures of compounds **17**–**30**.

Nine new C9 polyketides, named aspiketolactonol (**17**), aspilactonols A–F (**18**–**23**), aspyronol (**25**), and epiaspinonediol (**27**), together with five known polyketides, (*S*)-2-(2′-hydroxyethyl)-4-methyl-γ-butyrolactone (**24**), dihydroaspyrone (**26**), aspinotriol A (**28**), aspinotriol B (**29**), and chaetoquadrin F (**30**) (Figure 2), have been isolated from the secondary metabolites of *Aspergillus* sp. 16-02-1, which was isolated from a deep-sea sediment at a Lau Basin hydrothermal vent (depth 2255 m, temperature 114 °C) in the southwest of the Pacific Ocean. All of these compounds show strong cytotoxic activities against human cancer cell lines such as K562, HL-60, HeLa, and BGC-823 [42].

**Figure 3:**
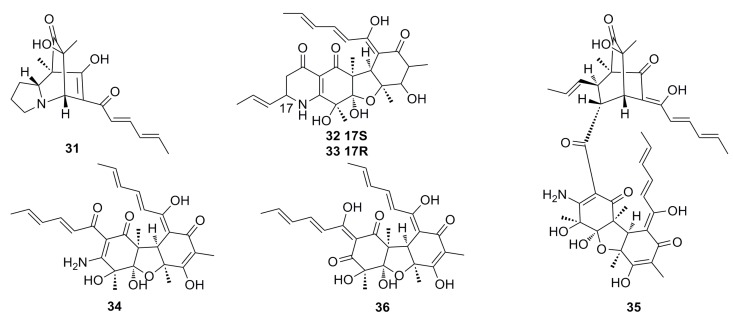
Chemical structures of compounds **31**–**36**.

Five new nitrogen-containing sorbicillinoids, named sorbicillamines A–E (**31**–**36**) (Figure 3), have been isolated from an agitated culture of a deep-sea fungus *Penicillium* sp. F23-2. The structures of **31** to **36**, including absolute configurations, were determined based on MS, NMR, and circular dichroism (CD) data. Unfortunately, all these compounds show weak cytotoxicity (IC_50_ > 10 μM) against the HeLa, BEL-7402, HEK-293, HCT-116, and P388 cell lines. Moreover, the strain F23-2 is able to produce indole alkaloids and terpenoids when it is cultured under static conditions [43].

**Figure 4:**
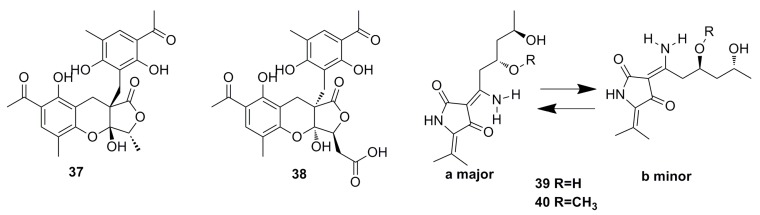
Chemical structures of compounds **37**–**40**.

Two highly oxygenated polyketides, penilactone A (**37)** and B (**38**) (Figure 4), containing a new carbon skeleton formed from two 3,5-dimethyl-2,4-diol-acetophenone units and a γ-butyrolactone moiety, have been isolated from a fungus named *Penicillium crustosum* PRB-2, which was derived from deep water environments of the Antarctic Ocean. Penilactones A and B possess antipodal absolute stereochemistry and show weak antitumor activity. However, penilactone A exhibits weak cytotoxic activity against NF-κB (40%) at the concentration of 10 mM [25].

Two new fungal hybrid polyketides, cladosin F (**39**) and G (**40**) (Figure 4), with a rare 6(3)-enamino-8,10-dihydroxy-tetraketide system have been discovered from the deep-sea-derived fungus *Cladosporium* *sphaerospermum* 2005-01-E3, guided by the OSMAC approach. Both exhibit weak *in vitro* antitumor activity [44].

#### 3.1.2. Steroid Derivatives

**Figure 5:**
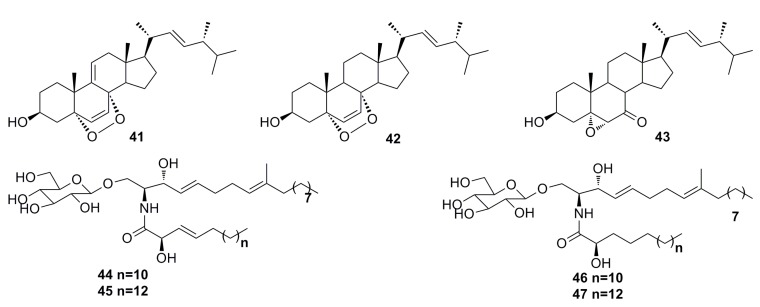
Chemical structures of compounds **41**–**47**.

Seven secondary metabolites including 9(11)-dehydroergosterol peroxide (**41**), ergosterol peroxide (**42**), (22*E*,24*R*)-5α,6α-epoxy-3β-hydroxyergosta-22-ene-7-one (**43**), and cerebroside A (**44**), B (**45**), C (**46**), and D (**47**) (Figure 5) have been described by Cui *et al.* in 2013 from the deep-sea fungus *Paecilomyces lilacinus* ZBY-1. These compounds exhibit cytotoxic activity against K562, MCF-7, HL-60, and BGC-823 cells with IC_50_ of 22.3 to 139.0 μM [45].

**Figure 6:**
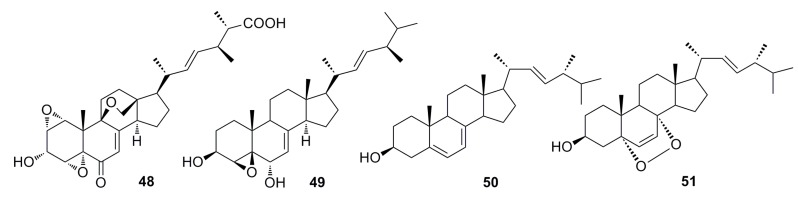
Chemical structures of compounds **48**–**51**.

Li *et al.* (2012) reported two sterols, sterolic acid (**48**) and sterol (**49**) (Figure 6), which were isolated from the crude extract of the deep-sea fungus *Penicillium* sp. metabolites. Compounds **48** and **49** show slight cytotoxic effects against MCF-7 cells and A549 cells [46].

**Figure 7:**
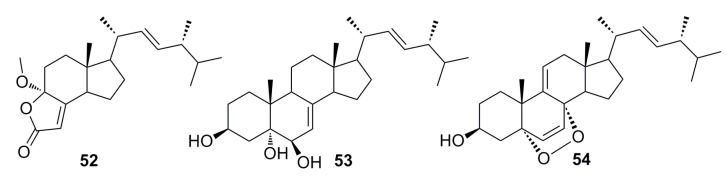
Chemical structures of compounds **52**–**54**.

Compounds ergosterol (**50**) and ergosterol peroxide (**51**) (Figure 6) have been isolated from the cultures of the deep-sea fungus *Penicillium* sp. F00120, which was collected from the northern South China Sea at a depth of 1300 m [47]. Compound **51** also has been isolated from the deep-sea-derived fungus *Aspergillus* sp. CXCTD-06-6a [48]. Both compounds show good cytotoxicity (27.2%–31.5%) against the HeLa cell line at 10 μM [47].

(17*R*)-17-Methylincistererol (**52**) and cerevisterol (**53)** (Figure 7) were isolated from the deep-sea fungus *Aspergillus sydowi* by a bioassay-guided method. Compound **52** has been considered as a highly degraded product of sterol, while compound **53** is a common metabolite of fungi. Both exhibit cytotoxicity [49]. Shang *et al.* (2012) [50] also reported compound **53** together with compound **54** (Figure 7), which was isolated from the deep-sea fungus *Penicillium commune* SD-118. All of them show a slight cytotoxicity against MCF-7 [50].

#### 3.1.3. Indole Derivatives

**Figure 8:**
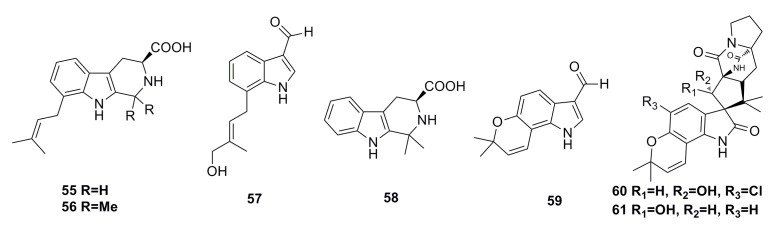
Chemical structures of compounds **55**–**61**.

Three new prenylated indole alkaloids, including two β-carbolines, penipaline A (**55**) and B (**56**), and one indole carbaldehyde derivative, penipaline C (**57**), as well as two known indole-derived analogs (**58**,**59**) (Figure 8), have been isolated from the metabolites of the deep-sea fungus *Penicillium paneum* SD-44, which was cultivated in a 500-L bioreactor. All these metabolites show cytotoxicity against A549 (IC_50_: 20.4–21.5 μM) and HCT-116 (IC_50_: 14.9–18.5 μM) cell lines [51].

5-Chlorosclerotiamide (**60**) and 10-epi-sclerotiamide (**61**) (Figure 8) are the secondary metabolites of the deep-sea fungus *Aspergillus westerdijkiae* DFFSCS013, which show excellent cytotoxicity against K562 cell line with IC_50_ of 44 μM and 53 μM, respectively [52].

#### 3.1.4. Sesquiterpenoids

**Figure 9:**
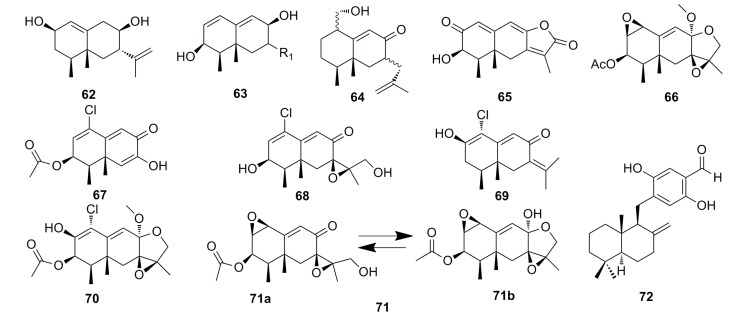
Chemical structures of compounds **62**–**72**.

Chemical investigation of the *Penicillium* sp. PR19 N-1 isolated from the Antarctic deep-sea has yielded five new eremophilane-type sesquiterpenes (**62**–**66**) (Figure 9). These compounds show strong cytotoxicity against HL-60 cells (IC_50_: 28.3–45.8 μM) and the A-549 cells (IC_50_: 5.2–82.8 μM) [53]. In 2013, Wu *et al.* [24] isolated a new chloro-trinoreremophilane sesquiterpene **67**, three new chlorinated eremophilane sesquiterpenes (**68**–**70**), and a known compound, eremofortine C (**71**) (Figure 9), from the same strain, PR19N-1. They found that all the compounds (**67**–**71**) exhibit cytotoxic activity against HL-60 and A549 cell lines. Particularly, compound **67** shows great cytotoxicity against HL-60 and A549 cell lines with IC_50_ of 11.8 and 12.2 μM, respectively [24].

In 2010, Chen *et al.* reported a new sesquiterpene, hydroquinone (**72**) (Figure 9), which was isolated from the metabolites of the deep-sea fungus *Phialocephala* sp. This compound displays strong cytotoxic activity against P388 and K562 cells with IC_50_ of 0.16 μM and 0.05 μM, respectively [54].

#### 3.1.5. Alkaloid Compounds

**Figure 10:**
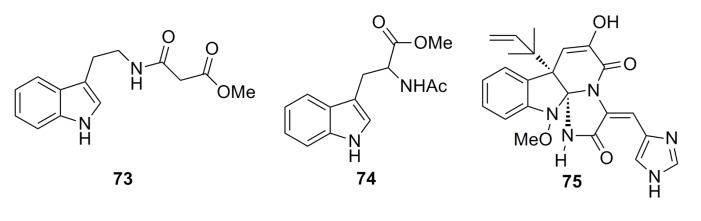
Chemical structures of compounds **73**–**75**.

Shang *et al.* (2012) reported three alkaloids (**73**–**75**) (Figure 10) that were isolated from the deep-sea fungus *Penicillium commune* SD-118. These alkaloid compounds show potent cytotoxicity against the DU145 cell line with IC_50_ of 4.3–5.0 μM. Moreover, compound **75** shows moderate cytotoxicity toward the HepG2, NCIH460, HeLa, and MDA-MB-231 cell lines with IC_50_ of 0.03, 0.05, 0.05, and 0.03 mM, respectively [50].

#### 3.1.6. Aromatic Compounds

**Figure 11:**
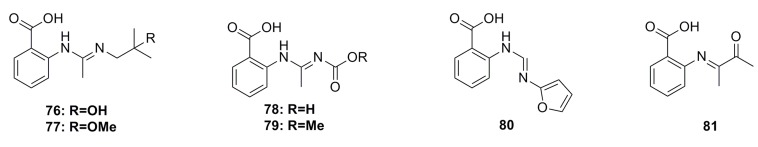
Chemical structures of compounds **76**–**81**.

Five new anthranilic acid derivatives, penipacids A–E (**76**–**80**), together with one known analogue (**81**) (Figure 11), which was previously synthesized, have been obtained from the ethyl acetate extract of the deep-sea fungus *Penicillium paneum* SD-44. These compounds exhibit cytotoxic activity against the RKO cell line (IC_50_: 8.4–9.7 μM). Especially, compound **81** displays remarkable cytotoxicity against the HeLa cell line with an IC_50_ of 6.6 μM [55].

**Figure 12:**
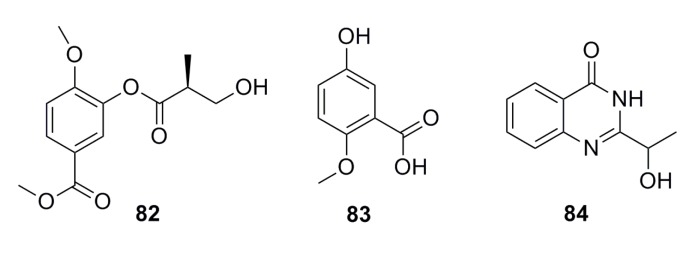
Chemical structures of compounds **82**–**84**.

A compound (2′*S*)-4-methoxy-3-(2′-methyl-3′-hydroxy) propionyl-methyl benzoate (**82**) (Figure 12) has been isolated from the metabolites of the deep-sea fungus *Aspergillus* sp. 16-02-1. This compound possesses 34.5%, 25.2%, 3.2%, and 15.5% cytotoxicity against human cancer cell lines K562, HL-60, HeLa, and BGC-823, respectively, at 100 μg/mL [56].

A secondary metabolite of 5-hydroxy-2-methoxy benzoic acid (**83**) (Figure 12) was isolated from the deep-sea fungus *Aspergillus* sp. CXCTD-06-6a, which shows 7.29% of cytotoxic activity against the HeLa cell line at 1.68 μg/mL [48].

An aromatic compound (**84**) (Figure 12) has been isolated from the metabolites of the deep-sea sediment-derived *Penicillium commune* SD-118. It exhibits moderate cytotoxicity against the SW1990 cell line with IC_50_ of 0.11 mM [50].

**Figure 13:**
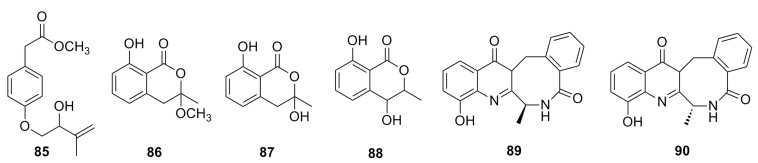
Chemical structures of compounds **85**–**90**.

A new methyl 2-(4-((2-hydroxy-3-methylbut-3-en-1-yl)oxy)phenyl) acetate (**85**), together with five known compounds (**86**–**90**) (Figure 13), has been isolated from the culture of the deep-sea fungus *Aspergillus westerdijkiae* SCSIO 05233. All of these display weak cytotoxic activities. Compound **89** displays weak antiproliferation activities towards K562 and promyelocytic HL-6 (IC_50_: 25.8–44.9 mM), while compound **90** shows strong antifouling activity with EC_50_ of 27.5 mM [57].

#### 3.1.7. Fatty Acids

**Figure 14:**
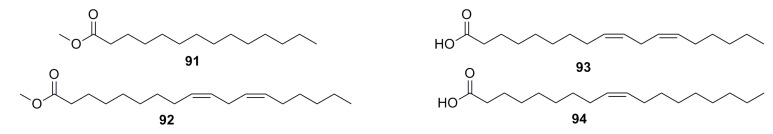
Chemical structures of compounds **91**–**94**.

Four fatty acid compounds, methyl myristate (**91**), methyl linoleate (**92**), linoleate (**93**), and oleinic acid (**94**) (Figure 14), have been isolated from the metabolites of the deep-sea fungus *Paecilomyces lilacinus* ZBY-1. All of them exhibit 30%–80% cytotoxicity against human cancer cell lines K562, MCF-7, HL-60, and BGC-823 at 100 mg/L [58].

#### 3.1.8. Pyrone Analogues

**Figure 15:**
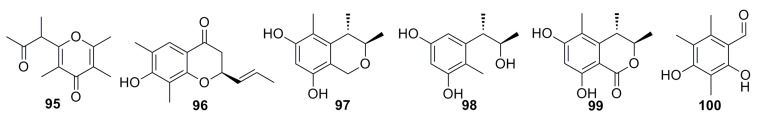
Chemical structures of compounds **95**–**100**.

Two new compounds, 2,3,5-trimethyl-6-(3-oxobutan-2-yl)-4*H*-pyran-4-one (**95**) and (2*R*)-2,3-dihydro-7-hydroxy-6,8-dimethyl-2-[(*E*)-prop-l-enyl]chromen-4-one (**96**), together with six known compounds (**97**–**100**) (Figure 15), were isolated from the deep-sea fungus *Aspergillus sydowi* by a bioassay-guided method. All these compounds exhibit various degrees of cytotoxicity. Compounds **96** and **100** show strong cytotoxicity against P388 cells with IC_50_ of 0.14 and 0.59 μM, respectively [49].

#### 3.1.9. Sorbicillin Derivative

**Figure 16:**
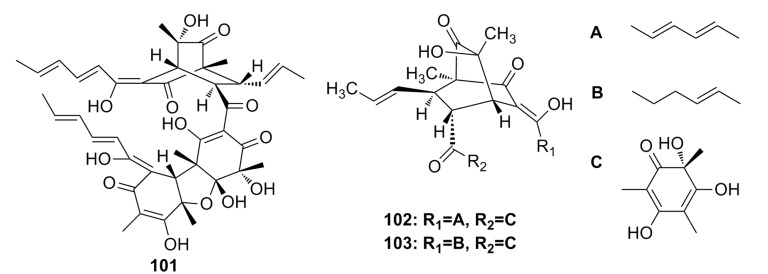
Chemical structures of compounds **101**–**103**.

A novel sorbicillin trimer, trisorbicillinone A (**101**) (Figure 16), was isolated from the deep-sea fungus *Phialocephala* sp. FL30r. Trisorbicillinone A shows cytotoxicity against P388 and HL60 cells with IC_50_ of 9.1 and 3.1 μM, respectively [59].

Two new bisorbicillinoids, named oxosorbiquinol (**102**) and dihydrooxosorbiquinol (**103**) (Figure 16), have been isolated from the deep-sea fungus *Phialocephala* sp. Both of them show cytotoxicity (IC_50_: 8.9–103.5 μM) against P388, A549, HL60, BEL-7402, and K562 cell lines [60].

#### 3.1.10. Breviane Derivative

**Figure 17:**
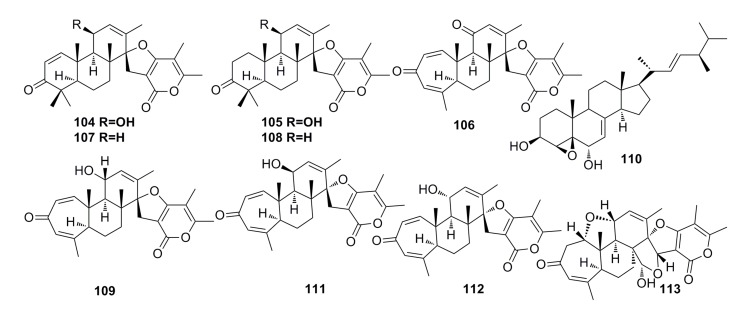
Chemical structures of compounds **104**–**113**.

Three new breviane spiroditerpenoids, breviones I–K (**104**–**106**), and the known breviones (**107**–**110**) (Figure 17) have been isolated from the crude extract of the deep-sea fungus *Penicillium* sp. and display strong cytotoxic effects against MCF-7 cells (IC_50_: 7.44–28.4 μM). Compound **106** exhibits cytotoxic activity against A549 cells with IC_50_ of 32.5 μM [46]. Similar compounds, Breviones F–H (**111**–**113**) (Figure 17), have been isolated from the same fungal species by Li *et al.* (2009) [61]. These compounds (**111**–**113**) show 25.2%–44.9% cytotoxicity against HeLa at 10 μg/mL. Particularly, compound **111** displays a very strong cytotoxicity to HIV-1 replication in C8166 cells with an EC_50_ of 14.7 μM [61].

#### 3.1.11. Compounds Containing Amino Acid Structure

**Figure 18:**
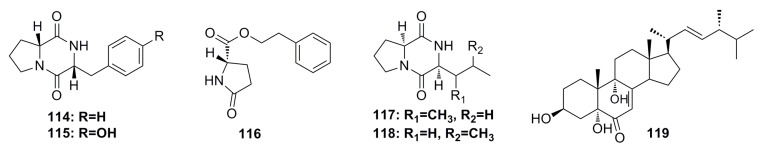
Chemical structures of compounds **114**–**119**.

Six metabolites, cyclo(d-Pro-d-Phe) (**114**), cyclo(d-Tyr-d-Pro) (**115**), phenethyl 5-oxo-l-prolinate (**116**), cyclo(l-Ile-l-Pro) (**117**), cyclo(l-Leu-l-Pro) (**118**), and 3β,5α,9α-trihydroxy-(22*E*,24*R*)-ergosta-7,22-dien-6-one (**119**) (Figure 18), have been isolated from the secondary metabolites of a mutated deep-sea fungal strain of *Aspergillus versicolor* ZBY-3. These compounds show certain cytotoxicity against K562 cells at a concentration of 100 μg/mL [62].

#### 3.1.12. Other Compounds

**Figure 19:**
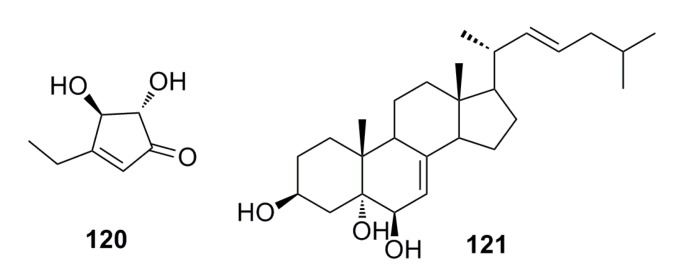
Chemical structures of compounds **120** and **121**.

A novel cyclopentenone, trichoderone (**120**), and a known compound, cholesta-7,22-diene-3b,5a,6b-triol (**121**) (Figure 19), were identified from the deep-sea fungus *Trichoderma* sp., which was isolated from the deep-sea sediment of the South China Sea. Compound **120** shows more than 80% cytotoxicity against A549 and NCIH460 cancer cell lines. The selectivity index for **120** was greater than 100. The two compounds also act as enzyme inhibitors against HIV protease and Taq DNA polymerase [18].

**Figure 20:**
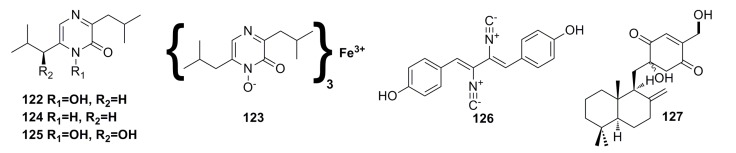
Chemical structures of compounds **122**–**127**.

New aspergillic acid (**122**), ferrineoaspergillin (**123**), aflatoxin (**124**), and (11*S*)-hydroxyl aspergillic acid (**125**) (Figure 20) have been isolated from the deep-sea fungus *Aspergillus* sp. 16-02-1. These compounds possess certain cytotoxicity against human cancer cell lines K562 (33.6%–43.6%), HL-60 (24.1%–53.3%), HeLa (18.8%–45.4%), and BGC-823 (36.2%–51.2%) at the concentration of 100 μg/mL [56].

A known antibacterial compound, xanthocillin X (**126**) (Figure 20), was isolated for the first time from the deep-sea fungus *Penicillium commune* SD-118. This compound displays not only remarkable antimicrobial activity against *Staphylococcus* *aureus* and *Escherichia coli* with MIC of 1–64 μg/mL, but also significant cytotoxicity against MCF-7, HepG2, H460, HeLa, DU145, and MDA-MB-231 cell lines (IC_50_: 7–22 μg/mL) [50,63].

One new sesquiterpene quinone, named penicilliumin A (**127**) (Figure 20), was isolated from the deep-sea fungus *Penicillium* sp. F00120. It inhibits *in vitro* proliferation of mouse melanoma (B16), human melanoma (A375), and human cervical carcinoma (HeLa) cell lines with IC_50_ of 0.08, 0.06, and 0.12 mM, respectively [47].

**Figure 21:**
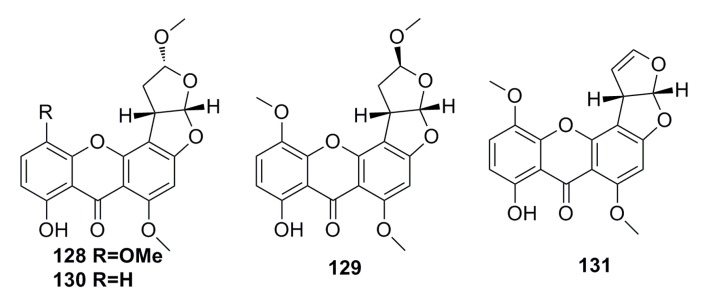
Chemical structures of compounds **128**–**131**.

Three new sterigmatocystin derivatives, oxisterigmatocystin A (**128**), oxisterigmatocystin B (**129**), and oxisterigmatocystin C (**130**), together with one known compound, 5-methoxysterigmatocystin (**131**) (Figure 21), have been isolated from the deep-sea fungus *Aspergillus versicolor*. The structures of the new compounds were elucidated by spectroscopic methods. All the compounds show cytotoxic activity against A549 and HL-60 cell lines, and compound **131** is the best with IC_50_ of 3.86 mM and 5.32 mM, respectively [64].

**Figure 22:**
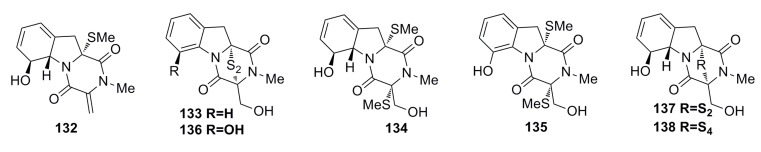
Chemical structures of compounds **132**–**138**.

Seven gliotoxin-related compounds including two new metabolites, bis(dethio)-10a-methylthio-3a-deoxy-3,3a-didehydrogliotoxin (**132**) and 6-deoxy-5a,6-didehydrogliotoxin (**133**), and five known metabolites (**134**–**138**) (Figure 22) have been isolated from the fungus *Penicillium* sp. strain JMF034, which was obtained from the deep-sea sediments of Suruga Bay, Japan. All these types of compounds exhibit significant cytotoxic activity against P388 (IC_50_: 0.02–3.40 μM), whereas compounds **136**–**138** containing a disulfide or tetrasulfide bond show potential inhibitory activity against histone methyltransferase (HMT) G9a (IC_50_: 2.1–6.4 μM) [65].

### 3.2. Antibacterial

Antimicrobials are the most important drugs to protect human beings from infective diseases. Deep-sea fungi are one of the potential pools for screening antimicrobial metabolites, which can be developed into new drugs.

#### 3.2.1. Prenylxanthones

**Figure 23:**
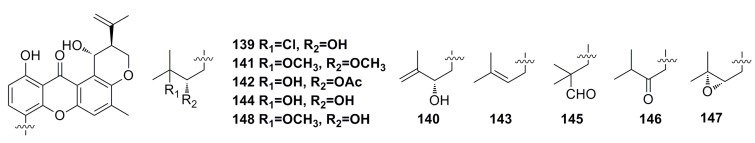
Chemical structures of compounds **139**–**148**.

Four new prenylxanthones, emerixanthones A–D (**139**–**143**), together with six known analogues (**144**–**148**) (Figure 23), were isolated from the culture of the deep-sea fungus *Emericella* sp. SCSIO 05240. All of them show weak growth inhibition against bacteria. The inhibition zone of compounds **139** and **141** against *Escherichia coli* (ATCC 29922), *Klebsiella pneumonia* (ATCC 13883), *Staphylococcus aureus* (ATCC 29213), *Enterococcus faecalis* (ATCC 29212), *Acinetobacter baumanii* (ATCC 19606), and *Aeromonas hydrophila* (ATCC 7966) is 1–3 mm in diameter. Moreover, compound **143** displays broad antifungal activities (3–4 mm in diameter) against *Fusarium* sp., *Penicillium* sp., *Aspergillus niger*, *Rhizoctonia solani*, *Fusarium oxysporium* f*.* sp. *niveum*, and *Fusarium oxysporium* f*.* sp. *cucumeris* [8].

#### 3.2.2. Depsidone-Based Analogues

**Figure 24:**
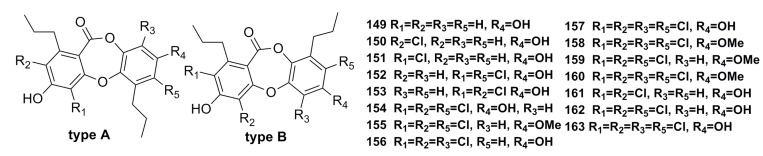
Chemical structures of compounds **149**–**163**.

Fifteen new depsidone-based analogues, named spiromastixones A–O (**149**–**163**) (Figure 24), have been isolated from the fermentation broth of the deep-sea fungus *Spiromastix* sp. These compounds exhibit significant growth inhibition (MIC: 0.1–8.0 μg/mL) against Gram-positive bacteria including *Staphylococcus aureus*, *Bacillus thuringiensis*, and *Bacillus subtilis*. In addition, compounds **154**–**158** display potential inhibitory effects on methicillin-resistant bacterial strains of *Staphylococcus aureus* (MRSA) and *Staphylococcus epidermidis* (MRSE). Moreover, compound **158** inhibits the growth of the vancomycin-resistant bacteria of *Enterococcus faecalis* and *Enterococcus faecium* (VRE) [66].

#### 3.2.3. Triple Benzene Compound

**Figure 25:**
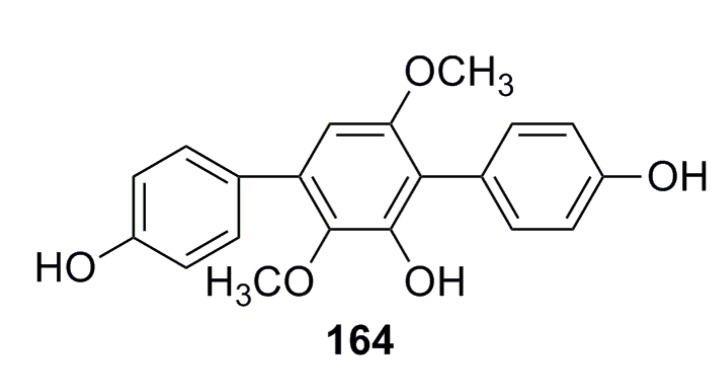
Chemical structure of compound **164**.

Liu *et al.* (2013) [67] have reported a compound (**164**) (Figure 25) that was isolated from the deep-sea fungus *Aspergillus candidus* and possesses broad and high antibacterial activity (inhibition: 83.9%–100%) against *Staphylococcus aureus*, *Bacillus subtilis*, *Vibrio* sp. 385, *Vibrio* sp. 333, and *Vibrio* sp. 1758 [67].

#### 3.2.4. Citromycetin Analogue

**Figure 26:**
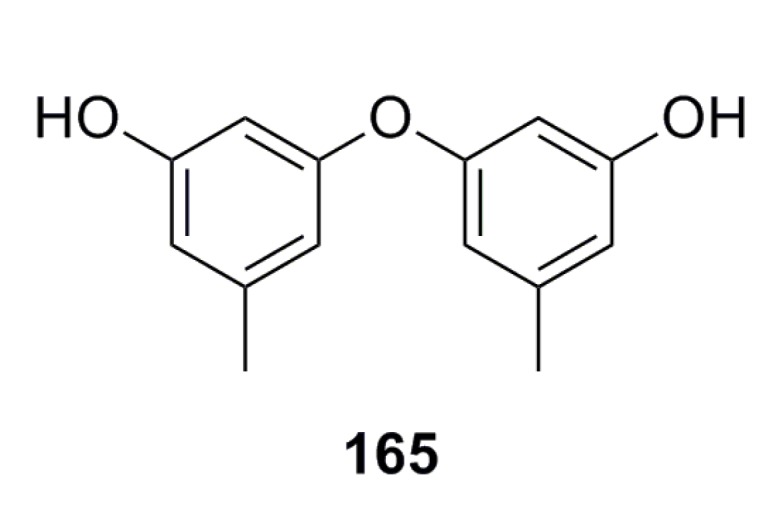
Chemical structure of compound **165**.

A new citromycetin analogue, diorcinol (**165**) (Figure 26), has been isolated from the metabolites of *Ascomycota* sp. Ind19F07, which was collected from the deep-sea sediment in the Indian Ocean. Based on *in vitro* tests, compound **165** shows strong antibacterial activity against both Gram-positive and -negative bacteria such as *Acinetobacter baumanii*, Escherichia coli, *Enterococcus faecalis*, and *Staphylococcus aureus* [68].

#### 3.2.5. Other Compounds

**Figure 27:**
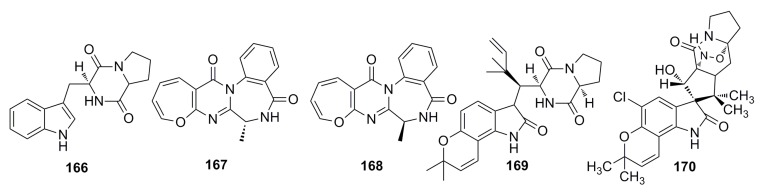
Chemical structures of compounds **166**–**170**.

Recently, Zhang *et al.* (2014) [23] have evaluated the antimicrobial activities of the secondary metabolites produced by eight novel deep-sea-derived fungal species, *Acremonium implicatum* DFFSCS001 (AI001), *Aspergillus westerdijkiae* DFFSCS013 (AW013), *Alternaria tenuissima* DFFSCS003 (AT003), *Cladosporium cladosporioides* DFFSCS016 (CC016), *Cladosporium sphaerospermum* DFFSCS019 (CS019), *Engyodontium album* DFFSCS021 (EA021), *Geomyces vinaceus* DFFSCS022 (GV022), and *Tritirachium* sp. DFFSCS034 (TS034). These fungal species were isolated from sediments of the South China Sea [23], and almost all the ethyl acetate extracts of the fungal species show strong antibacterial activity against two larval-settlement-inducing bacteria *Loktanella hongkongensis* and *Micrococcus luteus*, and one marine pathogenic bacterium*.* Based on bioassay-guided isolation technique, they have isolated five compounds (**166**–**170**) (Figure 27) from the extract of *Aspergillus westerdijkiae* DFFSCS013 that show antifouling activity against *Bugula neritina* larval settlement with an EC_50_ of 6.4–34.9 μg/mL [25].

### 3.3. Antiviral

#### Polyketides

**Figure 28:**
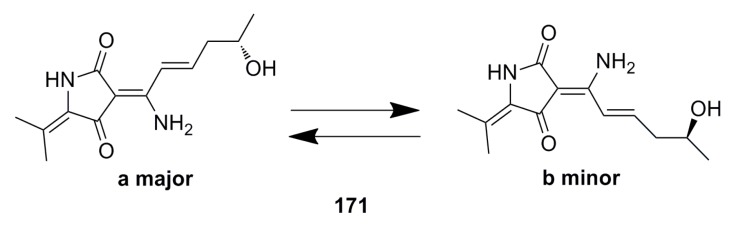
Chemical structure of compound **171**.

Although many polyketide compounds have been identified, the compounds derived from deep-sea fungi with antiviral activity are rare. Only one fungal hybrid polyketide with a new structure, cladosin C (**171**) (Figure 28), has been reported from the deep-sea fungus *Cladosporium sphaerospermum* 2005-01-E3. However, this compound shows good antiviral activity against influenza A H1N1 virus with an IC_50_ of 276 μM [9].

### 3.4. Antioxidant and Antifouling

#### 3.4.1. Diketopiperazine Derivatives

**Figure 29:**
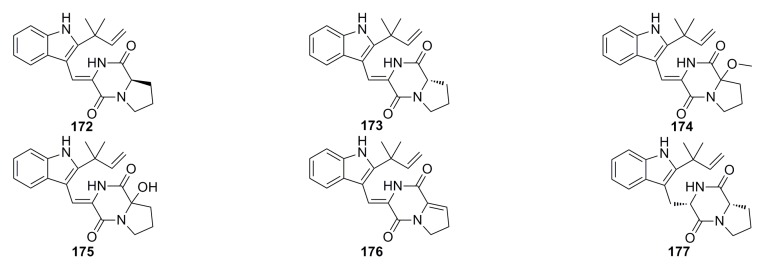
Chemical structures of compounds **172**–**177**.

Kong *et al.* (2014) [69] have reported a new diketopiperazine, brevianamide W (**172**), and five known diketopiperazine derivatives, diketopiperazine V (**173**), brevianamide Q (**174**), brevianamide R (**175**), brevianamide K (**176**), and brevianamide E (**177**) (Figure 29), which were isolated from the EtOAc extract of the deep-sea fungus *Aspergillus versicolor* CXCTD-06-6. These compounds exhibit moderate radical scavenging activity against DPPH with clearance ratios of 55.0% (**172** and **173**), 53.7% (**174**), 46.2% (**175**), 61.4% (**176**), and 19.3% (**177**) at 13.9 μM, respectively [69].

#### 3.4.2. Hydroxyphenylacetic Acid

**Figure 30:**
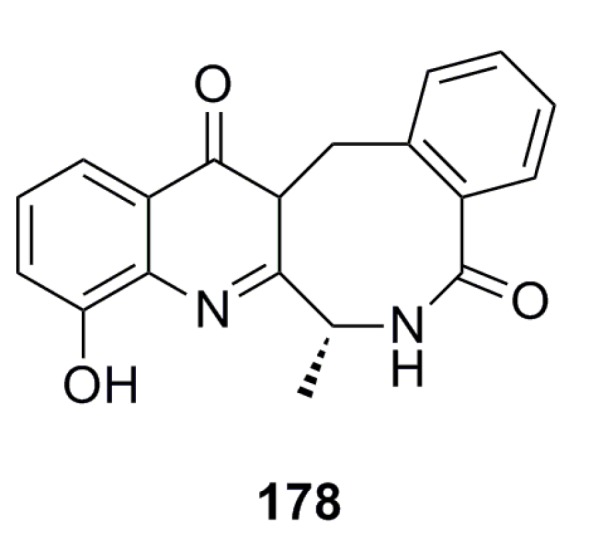
Chemical structure of compound **178**.

Compound **178** (Figure 30) has been reported from the deep-sea-derived fungus *Aspergillus westerdijkiae* SCSIO 05233 and shows strong antifouling activity with an EC_50_ of 8.8 mg/mL [57].

### 3.5. Antifungal

**Figure 31:**
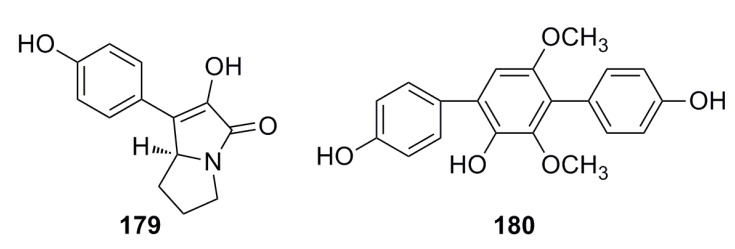
Chemical structures of compounds **179**–**180**.

One compound, *p*-hydroxyphenopyrrozin (**179**) (Figure 31), with a new structure has been isolated from the deep-sea fungus *Chromocleista* sp. and characterized on the basis of mass spectroscopy, NMR experiments, derivatization, and X-ray crystallography studies. The MIC of this compound is 25 μg/mL against *Candida albicans* [1]. In 2013, Liu *et al.* reported a compound (**180**) (Figure 31) that was isolated from the deep-sea fungus *Aspergillus candidus* and shows great growth inhibition against *Candida albicans* [67].

### 3.6. Compounds with Other Bioactivities

**Figure 32:**
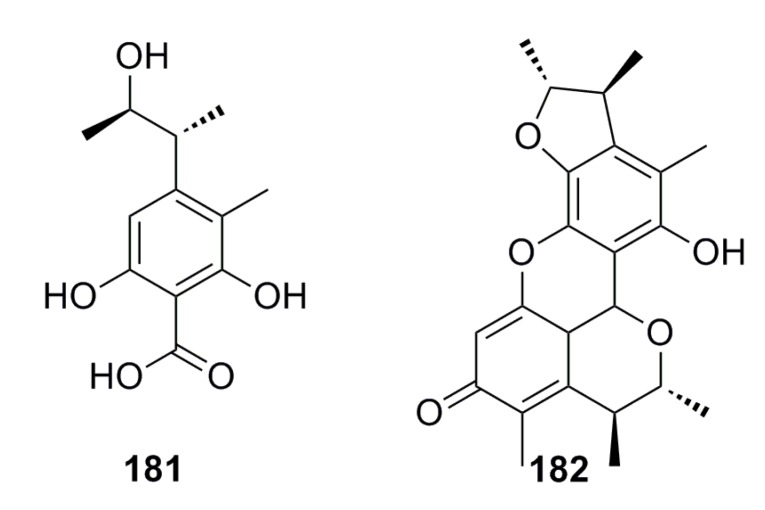
Chemical structures of compounds **181** and **182**.

Two citrinin type compounds, phenol A acid (**181**) and penicitrinone A (**182**) (Figure 32), with anti-Aβ peptide aggregation inhibition activity have been isolated from the deep-sea fungus *Aspergillus* sp. SCSIOW 3, which show Aβ_42_ assembling inhibition activity (40.3%–72.3%) at 100 μM [70].

## 4. Conclusions

The fungi in deep-sea environments are very diverse and abundant, making them a versatile reservoir of metabolites with both new structures and bioactivities that can be of potential use, acting as leading compounds to synthesize new modern medicine. Although the research on deep-sea fungi is not as up-to-date as the research on fungi in other environments such as terrestrial soil, fresh water, and shallow marine areas due to difficulties in both sample collection and fungal cultivation methods, more and more fungi have been cultivated from the deep sea based on culture-dependent methods. These deep-sea fungi can provide a potential source for natural bioactive product screening and new drug discovery. Up to now, more than 180 new and/or bioactive secondary metabolites from deep-sea fungi with broad bioactivities, such as anticancer, antimicrobial, antifungal, anti-larval settlement, and antiviral, have been described in the literature. Most of the investigated bioactive compounds exhibit cytotoxic activity, then antimicrobial activity. These bioactive compounds not only help deep-sea fungi to defend themselves against predators in the natural ecosystem, but also have the potential of becoming treatments for human diseases and probes for new biological targets. Work to isolate fungi from deep-sea environments and characterize their bioactive metabolites is underway and is of increased importance due to the urgent need for new drugs to overcome emerging and drug-resistant diseases.
